# Successful Operation for Stone in the Bladder

**Published:** 1875-08

**Authors:** E. O. Gratton

**Affiliations:** Hebron, Ill.


ARTICLE III.

### SUCCESSFUL OPERATION FOR STONE IN THE BLADDER.

BY E. O. GRATTON, M.D., HEBRON, ILL.

On Tuesday, Feb. 23d, 1875, I was called to see Mrs. Betsey Lovejoy, of Genoa Junc., Wisconsin, aged about 43 years. Found her suffering with stone in the bladder.

Called twice between that and Saturday, the 27th, at which time I performed the operation of lithotomy, with the assistance of Dr. Woodruff, of Harvard, Ill. The stone, weighing five and one-half drachms, was taken out by dilatation, with cutting about two lines of the external sphincter.

The patient is, at this date, July 13th, as well as before the growth commenced, following her usual avocation, which is that of a seamstress. She has had no trouble to retain the urine from the first.

